# Hemostasis of small-intestinal diverticular bleeding with the over-the-scope clip method

**DOI:** 10.1055/a-2098-8596

**Published:** 2023-08-21

**Authors:** Hiroshi Tanabe, Koichiro Kawano, Reiko Kawano, Takao Katoh, Katsuhisa Nishi, Yoriaki Komeda, Mamoru Takenaka

**Affiliations:** 1 Department of Gastroenterology, Hyogo Prefectural Awaji Medical Center, Sumoto, Japan; 2 Department of Gastroenterology and Hepatology, Kindai University Faculty of Medicine, Osaka-Sayama, Japan

Effective endoscopic hemostatic techniques for small-intestinal diverticular bleeding have not been established. While endoscopic band ligation (EBL) is reported to be effective for colonic diverticular hemorrhage, it has been reported to cause delayed perforation owing to a completely occluded ligature site and necrosis ( Fig. 1 ) 1 . Reports of EBL in the small intestine suggest that the risk of perforation may be high because the ligature site is often ligated down to the serosa 2 . Therefore, a safe and effective hemostatic method for small-intestinal diverticular bleeding is required. It has been reported that application of an over-the-scope (OTS) clip ( Fig. 2 ) can be useful for colonic diverticular hemorrhage 3 4 , but there have been no reports of their use for small-bowel diverticular bleeding.

**Fig. 1:**
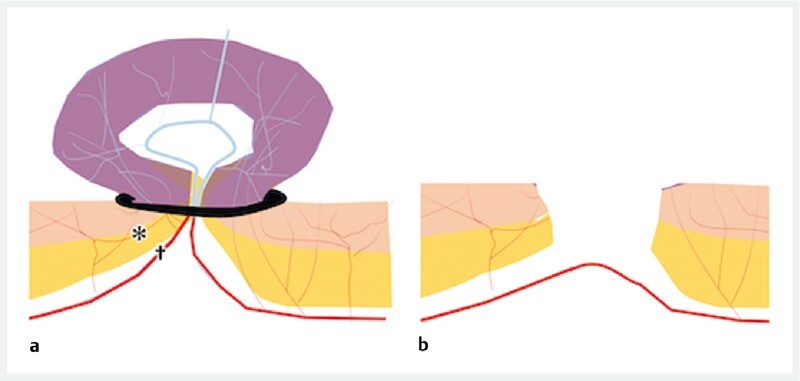
Schema of endoscopic band ligation (EBL) showing: **a** in standard EBL, the ligatured area covers the entire circumference, meaning that not only the responsible vessel (†) but also the surrounding vessels (*) are ligated; **b** the sutured mucosa can subsequently become necrotic and desquamate to form an ulcer, which may perforate if it does not heal properly.

**Fig. 2:**
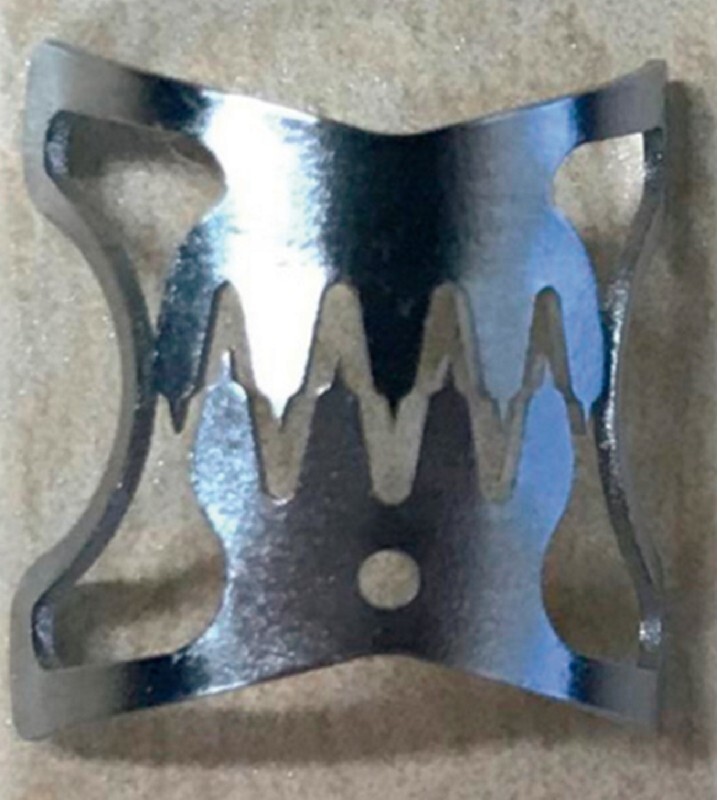
Photograph of an over-the-scope clip (Ovesco Endoscopy AG, Germany).

A 56-year-old woman presented to our hospital with gastrointestinal bleeding. A diverticulum with blood clots was detected at the distal end of the ileum on lower gastrointestinal endoscopy. No bleeding was observed after the clots had been removed, but active bleeding was observed after stimulation with a nontraumatic tube 5 . Therefore, this diverticulum was determined to be the source of the bleeding, and hemostasis was attempted using OTS clipping. The scope was removed after a marking clip had been placed near the diverticulum, and an OTS clip was attached to the scope, which was then reinserted. The diverticulum was then inverted and sutured using the OTS clip, and hemostasis was achieved ( Fig. 3 ; Video 1 ). No rebleeding or perforation occurred thereafter.

**Fig. 3:**
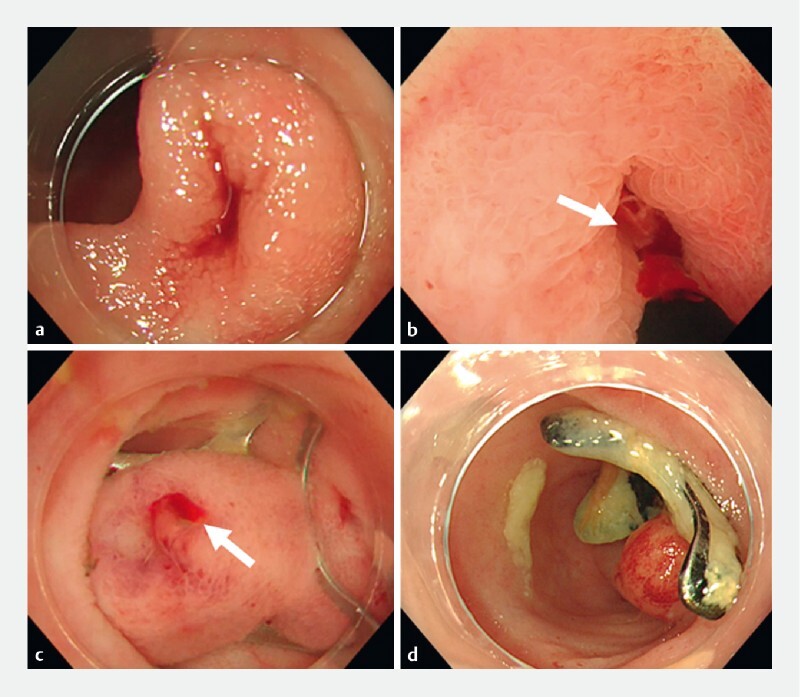
Endoscopic images of the application of an over-the-scope (OTS) clip for small-intestinal diverticular bleeding showing: **a** a diverticulum with blood clots at the distal end of the ileum; **b** active bleeding after stimulation with a non-traumatic tube, even though no bleeding was observed after removing the clots; **c** the OTS clip applied to the diverticulum and released with suction; **d** congestion of the sutured mucosa but with the OTS clip still in place at follow-up endoscopy 3 months post-treatment.

**Video 1** Hemostasis of small-intestinal diverticular bleeding is achieved with the over-the-scope clip method.


With the use of an OTS clip, the ligation site is not completely occluded ( Fig. 4 ) and, although congestion is seen, necrosis and desquamation are not observed, suggesting there is a low risk of perforation. To our knowledge, this is the first report of hemostasis being achieved for a diverticular hemorrhage in the small intestine using an OTS clip.

**Fig. 4:**
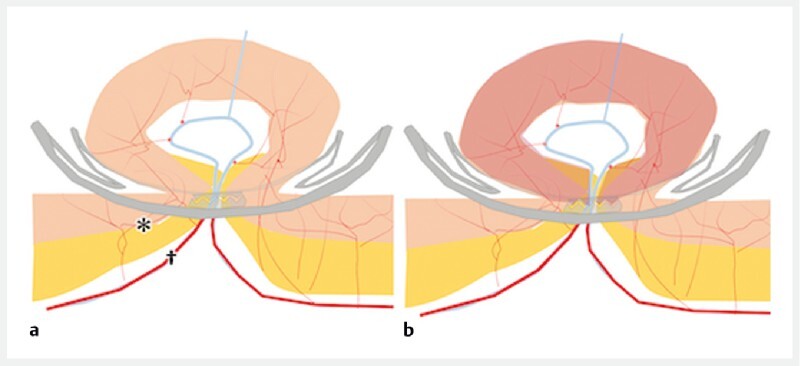
Schema of the over-the-scope (OTS) clip method of ligation showing: **a** how the OTS clip blocks blood flow only in the area where the teeth meet, meaning the responsible vessel (†) is ligated, but blood flow is maintained in the surrounding vessels (*); **b** the sutured mucosa is congested but not dislodged, and the OTS clip remains in place.

Endoscopy_UCTN_Code_TTT_1AQ_2AZ

## Notes

Endoscopy E-Videos

https://eref.thieme.de/e-videos
*E-Videos* is an open access online section of the journal *Endoscopy* , reporting on interesting cases and new techniques in gastroenterological endoscopy. All papers include a high-quality video and are published with a Creative Commons CC-BY license. Endoscopy E-Videos qualify for HINARI discounts and waivers and eligibility is automatically checked during the submission process. We grant 100% waivers to articles whose corresponding authors are based in Group A countries and 50% waivers to those who are based in Group B countries as classified by Research4Life (see: https://www.research4life.org/access/eligibility/ ). This section has its own submission website at https://mc.manuscriptcentral.com/e-videos

## References

[1] Sato Y Yasuda H Fukuoka A Delayed perforation after endoscopic band ligation for colonic diverticular hemorrhage Clin J Gastroenterol 2020 13 6 10 31367844 10.1007/s12328-019-01027-0

[2] Barker K B Arnold H L Fillman E P Safety of band ligator use in the small bowel and the colon Gastrointest Endosc 2005 62 224 227 16046983 10.1016/s0016-5107(05)00557-2

[3] Probst A Braun G Goelder S Endoscopic treatment of colonic diverticular bleeding using an over-the-scope clip Endoscopy 2016 48 01 E160 27116098 10.1055/s-0042-106167

[4] Kawano K Takenaka M Kawano R Efficacy of over-the-scope clip method as a novel hemostatic therapy for colonic diverticular bleeding J Clin Med 2021 10 2891 34209655 10.3390/jcm10132891 PMC8268121

[5] Kawano K Takenaka M Kawano R Nontraumatic tube method for detecting a vessel responsible for colonic diverticular hemorrhage Endoscopy 2022 54 E240 E241 34102676 10.1055/a-1486-6476

